# The role of social networks for combating COVID-19 pandemic: a study with reference to the Chinese new immigrants in Germany

**DOI:** 10.1186/s41257-023-00083-2

**Published:** 2023-03-21

**Authors:** Qian Zhu, Ming Lu, Yanjia Qin

**Affiliations:** 1 School of Sociology and Anthropology, Sun Yat-Sen University, Blk410135 Xinggang Xi Road, Guangzhou, 510275 No China; 2 Department of Social and Cultural Anthropology, University of Cologne, Cologne, Germany; 3 School of Ethnology and Sociology, Yunnan University, Kunming, China

**Keywords:** Chinese new immigrants (*xinyimin*), Social networks, COVID-19 pandemic, Germany

## Abstract

Social network theories are used extensively to analyze the international migration of Chinese to overseas regions in the era of Market Economy Reform since 1978. Attention is paid especially on the role of social networks among overseas Chinese on disaster relief in China. Focusing on the ongoing COVID-19 pandemic, this paper investigates how social networks work as a crucial mechanism through which Chinese immigrants in Germany initiated and delivered monetary and material donations to China in early 2020 and then organized self-help in their everyday lives in Germany in late 2020. Different from previous studies, this paper scrutinizes social networks for disaster relief on the macro, meso, and micro levels. Multi-site ethnographic fieldwork in China and Germany combined with online and offline data collected from focus group sessions, interviews with individuals, participant observations, surveys, analysis of news reports on the pandemic, and analysis on relevant policies are utilized comprehensively to collect data on the three levels. This research discovers that internet tools – represented by WeChat – have integrated tightly into the traditional social networks of Chinese immigrants and consolidated the cultural cohesion from overseas Chinese to their connections in China. This paper aims at contributing to present studies on Chinese new immigrants, social network, and disaster management theories with an updated ethnographic case on the COVID-19 pandemic from Germany.

## Introduction

The on-going COVID-19 pandemic has affected people’s everyday lives all over the globe in the past three years. The outbreak of COVID-19 in 2019 placed China in a vulnerable situation earlier than any other regions. After the first outbreak in Wuhan in Hubei province near the end of 2019, the pandemic soon spread nationwide and resulted in a public health crisis in the first half of 2020. Soon after that, the global spread of COVID-19 profoundly impacted many other countries and populations in the second half phase of 2020. Social networks are an important social capital for disaster relief. Chinese new immigrants have strong cultural, economic, and emotional connections with China and they have supported China in various disaster events in the past three decades. Taking Chinese new immigrants in Germany as a case, this research examines the roles of social networks among Chinese new immigrants when they combated COVID-19 pandemic and reveals their capacities on disaster relief.

### Literature review

#### Chinese new immigrants since 1978

The research on Chinese overseas since 1978 yields many findings. Studies prove that immigration is changing China (Pieke et al. 2019). The main body of Chinese overseas has transformed. On the one hand, the ethnic Chinese in Southeast Asia – those who were regarded as the main body of overseas Chinese and contributors to China – are the third or further generation, their connections to China are more emotional attachments (Thunø 2007). This indicates that strong ties to China are fading away. Meanwhile, a tremendous number of Chinese migrated to developed countries such as the US and European countries since the economic reforms initiated in 1978. They hold much closer ties to China than those living in Southeast Asia because most of them are the first generation of migrants. As the number increases every year, this group of people has become the main body of Chinese overseas in this era (Thunø 2007). On the other hand, China faced a new international environment when it opened its doors to the rest of the world. Chinese officials are fully aware of the situation and the importance of the support from overseas Chinese. According to Wang (2019),

“China is not offering its own version of universalism; instead, in the 18^th^ Party Congress, the message was that of finding its own road to modernity. That road is centered on two pillars. Within the country, national sovereignty can be made safe only through social order and continued development, something only a united and well-organized party-state can guarantee. Outside, security is the goal. The situation here is more complex, and its leaders know that there are many factors in the interdependent world that are beyond China’s control.”

New policies regarding Chinese overseas are adjusted accordingly. Xu You-sheng (2018) – the director of the overseas Chinese Affairs Office of the people’s Republic of China – points out the role of Chinese overseas in formulating the security of China in the new era. A new term was thus invented. The State Council expressed the need for a refocusing on “new migrants”.

“People who have left China’s Mainland to reside abroad are currently becoming an important rising force within overseas Chinese and ethnic Chinese communities. In the future, they will become a vital force friendly to us in the United States and some other developed Western countries, especially all kinds of overseas students who have settled locally” (Thunø 2001, 922).

“Chinese new immigrants” (*xinyimin*) is thus used as a term referring particularly to the Chinese migrating from China’s Mainland to other countries after the 1978 economic reform (Suryadinata 2017). They are the focus of the overseas Chinese in this study. These people include those who have obtained a foreign passport, those who hold a passport of the People’s Republic China but normally live in overseas countries, and Chinese students who are currently studying abroad.

The latest studies focusing on Chinese new immigrants investigate the identity, belonging, and integration of the Chinese new immigrants in their host countries (e.g., Zhang 2021). Suryadinata (2017) argues that “The nature of the new migrants is less “permanent” than previous waves of migrants”. They hold closer ties to their home country, China, because they cannot integrate into the overseas countries well. “Many new migrants are less well integrated into their adopted countries as the ease of communication and transportation in today’s globalized world allows them to maintain their links with their country of birth and retain a migrant mentality […]. This failure or inability to integrate into their host societies serves to perpetuate the old stereotype of ‘once a Chinese, always a Chinese’ (ibid.: 9–12)”. However, how do new immigrants organize themselves when they cannot well integrate into the host societies? Social networks play a persistent role in constructing the identity of new immigrants.

Social networks at the micro level are interpersonal connections. The ties of kinship, friendship, acquaintances, and colleagues connect new immigrants living in a foreign country and culture. Social networks at the meso level establish diverse associations. Associations become the key method to facilitate, sustain, and expand social networks of immigrants within associations and beyond. Members have strong ties within a certain association. The leaders of an association are standing in a structural hole between different members and relations with other associations and organizations. At the macro level, the nation state has its networks with its international migration worldwide through institutions and numerous associations. The state encourages new immigrants to establish associations based on their places of origin or professional associations, which have contributed to the establishment of immigrants’ sub national identity (Zhuang 2020). In 2008, the 15^th^ General Assembly of the European Federation of Overseas Chinese Associations was held in Berlin and it proposed: “at present, the total number of overseas Chinese (*huaqiao*) and ethnic Chinese (*huaren*) in Europe is about 2.5 million, there are more than 800 overseas Chinese organizations, and there are more than 43,000 Chinese restaurants”(Wang & Liu 2011). In recent years, the number of Chinese associations has been increasing in Europe, such as the case in Germany. Catering businesses is the major economic pillar for xinyimin in Europe.

A social network addresses social structural connections of diverse social actors (Scott 2000). It also highlights the productive consequences of social interactions and social conjunctions of actors. The result of the interactions and conjunctions is the accumulated social capital. Social capital “is the sum of the resources, actual or virtual, that accrue to an individual or a group by virtue of possessing a durable network of more or less institutionalized relationships of mutual acquaintance and recognition” (Bourdieu & Wacquant 1992). “Just as physical capital and human capital facilitate productive activity, social capital does as well” (Coleman 1988). Coleman highlights the feature of social groups and social network of social capital (Coleman 1990). Massey also argues that “People gain access to social capital through membership in networks and institutions and then convert it into other forms of capital to improve or maintain their position in society.” Strong ties and weak ties are two major connections of social networks for a social actor (individual or group). “Emphasis on weak ties lends itself to discussion of relations between groups and to analysis of segments of social structure not easily defined in terms of primary groups”.

Existing literature has explored the crucial function of social networks in the processes of migration (Massey 1987 and Portes 1995). Massey (2005) states the key characteristics of the social network of immigrants, including reciprocity exchanges, bounded solidarity, enforceable trust. All the forms of social capital are incorporated into social networks (Portes & Sensenbrenner 1993). Institutionalized relationships have been labeled as the establishment of migration associations in international migration. Thus, migration networks can be categorized into interpersonal networks and institutional networks (associations). Institutional networks can be further identified as internal system (the relationship among group members) and external system (relationship between associations, between associations and nation states or other international associations) (Li 1995; 1999b).

Previous empirical studies on Chinese international migration have documented various types of social networks, such as kinship networks (Choo 1968; Watson 1975; Li 2005), kinship-based or regional based networks contribute to the international migration of Wenzhou people in Zhejiang Province, China to Europe (Li 1999a; Wang 2002; Chen 2015). Chinese associations have been considered as the central actors of Chinese immigrants. Tan’s study on Chinese associations in Mauritius and Trinidad enables us to see “the nature and changing roles of the overseas Chinese associations in general, covering mutual aids, networking and transnational connection especially with China” (Tan 2016). The development and frequent interactions of overseas Chinese associations have been observed by some scholars (Liu 1998; Li 2015; Zhuang 2020). Zhuang (2020) argues that, “In the last 40 years, a large number of new Chinese emigrants went abroad, and they were most passionate in building various commerce chambers and fellow townsmen associations as mutual aid platforms and bridges to keep their relations with China”. The relationship between overseas Chinese and China is also explored by Thunø (2001), Tan (2007) and Suryadinata (2017).

Most research deals with the social network of Chinese new immigrants in a daily situation. Less emphasis is paid to a dynamic of social networks of Chinese new migrants in a disaster crisis. The disaster response capacity of different social groups has become a key element to deal with a crisis (Button 1993; Olsson & Folke 2003; Klein et al. 2003). Improving the ability to cope with disasters through social networks is an important aspect for disaster relief (Newman & Dale 2005). Risk is a kind of relational order, and risk identification is constructed by a set of cultural systems such as the institution, power, knowledge and practice of social relations (Boholm 2003). Meanwhile, the current empirical studies on the impact of COVID-19 pandemic focus either on Chinese immigrants (Wang et al. 2021) or China’s domestic coping strategies. Therefore, there is a need to link Chinese immigrants and China together in order to obtain a better picture of social networks of Chinese new immigrants. This will contribute to further explore the development of social capital of immigration.

#### Disaster studies, Chinese new immigrants and their donations to China

The United Nations Office for Disaster Risk Reduction (UNISDR) defines a disaster as “a serious disruption of the functioning of a community or a society involving widespread human, material, or environmental losses and impacts which exceeds the ability of the affected community to cope using only its own resources” (UNISDR 2009). Coping capacity is defined as “the ability of people, organizations and systems, using available skills and resources, to face and manage adverse conditions, emergencies or disasters” (ibid. 2009). “The goal of disaster risk reduction programmes is to reduce disaster risks by building capacity and increasing the resilience of communities at risk, thus enhancing their security and wellbeing” (Niekerk 2011). International organizations are established in order to reduce disaster risk, such as the United Nations Office for Disaster Risk Reduction (UNISDR). Slobodeniuc believes that “each state was encouraged to engage the relevant national scientific and technological communities and to mobilize the necessary human and financial support for the achievement for international actions on the prevention and reduction of natural disasters” (Slobodeniuc 2012).

Disasters occur in specific international and social-cultural contexts (Susman et al. 1983). Disaster adaptation and relief is a process of cultural adaptation; it also has local and group characteristics (Blaikie et al. 2003). According to Oliver-Smit (1996), “Recent perspectives in anthropological research define a disaster as a process/event involving the combination of a potentially destructive agent(s) from the natural and/or technological environment and a population in a socially and technologically produced condition of vulnerability. From this basic understanding three general topical areas have developed: (a) a behavioral and organizational response approach, (b) a social change approach, and (c) a political economic/environmental approach”.

In the last three decades, Chinese new immigrants have played an important role in fund-raising and donations during the period of various disasters in China. During the SARS epidemic in 2003 (Chao 2004) and the Wenchuan earthquake in 2008, Chinese new immigrants organized spontaneously to donate money and materials which contributed to China’s disaster relief (Suryadinata 2017). Most of the existing literature focuses mainly on daily donations of Chinese new immigrants on various causes in China (See e.g., Zhou and Li 2016). Less attention has been paid to the donations from Chinese new immigrants in emergencies, e.g., for disaster relief. This leaves a gap in research on the relationship between xinyimin and China from the perspective of disaster management, in particular, how do xinyimin apply its social networks to coping with the pandemic crisis in different phases and what consequences of the interactions and connections. The COVID-19 pandemic provides a good chance to explore the reciprocity, interactions and connections between Chinese new immigrants and China over a period of time (more than one year in this study). Our study aims to explore this process and fill in this gap. The role of Chinese new immigrants on disaster relief have been well discussed by revealing the significant donations from xinyimin, but the function and dynamic of their social networks have not been well explored. This article extends to illustrate various levels of xinyimin’s social networks.

#### Studies on Chinese new immigrants in the era of WeChat

Chinese people’s everyday lives have been fundamentally changed by the development of the internet in the past decades. The internet provides “new tools of connectivity, information diffusion, and attention, which help citizens better connect, express ideas, organize, and mobilize” (Han 2018). The best example to understand the power of the internet in the ordinary lives of people in China is to examine the usage of WeChat. WeChat is the most popular mobile app for instant online communication in China. It was invented by Tencent in October 2010 (Chao and Mozur 2012). It had over one billion monthly active users in 2018 (TechNode 2018). It has become an essential component of Chinese people’s everyday life because it is estimated that on average Chinese users spend one-third of their online time using WeChat and they return to the app ten times or more every day (CitizenLab 2016). Ju et al. (2019) finds that WeChat, as a digital intermediary space, provides Chinese vulnerable mobile groups who cross the border between Zhuhai city and Macau with social solidarity, social interaction, information exchange, economic interest and other benefits (Ju et al. 2019).

### Research questions

The issues discussed above shed light on the following questions. What is the impact of the COVID-19 epidemic on the lives and work of Chinese new immigrants? How do they respond to the outbreak of COVID-19 pandemic in China? When the COVID-19 spread in the communities where they lived, how can the Chinese new immigrants conduct self-help activities to improve their capacity of coping with disasters? What kinds of social networks do the Chinese new immigrants have during the two phases of combating COVID-19 pandemic? What is the difference between social networks, organizations, and communication mechanism against pandemics comparing with previous disasters? Taking Chinese new immigrants in Germany as an example, this paper contributes to a deeper understanding the social networks of xinyimin and its transformation regarding the pandemic by answering these questions. This paper also aims to enrich the literature in the domains of Chinese new immigrants’ social networks and social capital, China’s disaster management, and virtual communities based on the development of information technology.

#### Research methods and multi-site ethnographic fieldwork

This paper answers these questions utilizing multiple research methods including interviews with individuals, focus-group sessions, online surveys, participant observations, and archive analysis of news reports and policy analysis. Multi-site ethnographic fieldwork was conducted in several sites in Germany and China as well. In Germany, forty-two interviews were conducted including semi-structured and non-structured interviews from March 2020 to March 2021. One hundred and ten interviewees in total participated in the interviews, including Chinese students, housewives, chefs, restaurant owners, leaders of various Chinese associations, refugees, volunteers of health-kit distribution organizations in different cities, journalists, officials, authors’ German friends, German colleagues, German classmates, and German landlords. Six focus-group sessions were conducted. The focus-group sessions covered Chinese new immigrants taking on various occupations, including leaders of Chinese associations, businesspersons, students, housewives and officials etc. All the interviewees are anonymized. Regarding online surveys, the research team designed and distributed questionnaires in Mandarin using Google Surveys between March 16 and April 26, 2020 in Germany. The survey was disseminated through WeChat and Facebook to Chinese new immigrants living in Europe – including WeChat friends, Moments (Pengyouquan), and groups.^1^ The survey finally reached 591 respondents living in Germany, Italy, and other countries with the majority (87.25%) living in Germany. Regarding participant observations, two authors of this paper are Chinese students living in Germany. One author has been actively engaged in the activities among Chinese new immigrants in the past five years, such as working as a secretary in Chinese associations. Therefore, the interviews, surveys, and participant observation cover the opinions and experience of Chinese new immigrants living in several cities in Germany, including Cologne, Bonn, Frankfurt, Munich, and Heidelberg.

The research team also conducted participant observations. For instance, we distributed health kits to Chinese students in Germany. We also participated in an event which face masks donated from China were sent to the hospital at the University of Cologne. The authors of this paper experienced the pandemic in both Germany and China because they travelled several times between China and Germany from January 2020 to 2022. They have lived in both two countries during the pandemic and they are familiar with people’s everyday lives in both of these pandemic-affected countries.

In China, three structured interviews were completed with the government officials in Wenzhou of Zhejiang province and Jiangsu province. These two places were selected because the governments were active engaging Chinese new immigrants during the pandemic. Wenzhou is one of the key hometowns of new immigrants in China. According to the survey of the city in 2014, there are about 700,000 Wenzhouese living abroad and in Macau and Hong Kong (Zhang 2011). The majority of Chinese overseas of Wenzhou origin belong to the group of new immigrants because they emigrated abroad after 1978, of whom more than fifty percent left China after 2000. Wenzhou was another severely affected city besides Wuhan in Hubei province. Thus, new immigrants originating in Wenzhou were the most active group of Chinese new immigrants who provided tremendous support to their hometown during the pandemic. Jiangsu is a province neighbor of Zhejiang. It has been strengthening its connections with Chinese new immigrants by encouraging them to build more associations in overseas countries. One interview was conducted online via WeChat. The other two interviews were conducted in Wenzhou where the interviewers could understand the working environment of government officials in China. The research yielded detailed insights into the pandemic and donations among Chinese new immigrants. We explore in the following section how China can mobilize Chinese new immigrants to support the anti-epidemic campaign inside China through networking.

## Findings and discussions

### Chinese new immigrants in Germany in the 2020s

The number of Chinese new immigrants in Germany has been significantly increasing in the past four decades. According to Zhuang (2001), the number of xinyimin in Germany was twenty thousand (20,000) in 1981, seventy thousand (70,000) in 1992, and one hundred and ten thousand (110,000) in 1999. In 2020, there were about two hundred thousand (200,000) Chinese in Germany (Wang et al. 2011).^2^ According to Giese (2003), instead of forming a Chinatown, Chinese immigrants are scattered throughout Germany, and have formed economic and employment characteristics dominated by family restaurants and service industries. The statistics show that about 24% of Chinese works and lives in Nordrhein-Westfalen, while 16.3% of them stay in Bayern and about 15% in Baden-Württemberg in 2020. It means that the Chinese somehow prefer to gather in some states in Germany. Leung (2004) and He (2012) explore the catering industry of Chinese migrants in Germany, and they highlight how the family ties or ethnic networks have shaped the Chinese entrepreneurship and the ethnic groups. Moreover, according to the Federal Statistical Office, approximately 70,000 Blue Card holders were on the Central Register of Foreigners at the end of 2021 (Statistisches-Bundesamt 2022). There were about 4200 Chinese holding the Blue Card which occupies 6% of the total Blue Card holders in Germany in 2021 (Federal office for Migration and Refugees 2022).

**Fig. 1:**
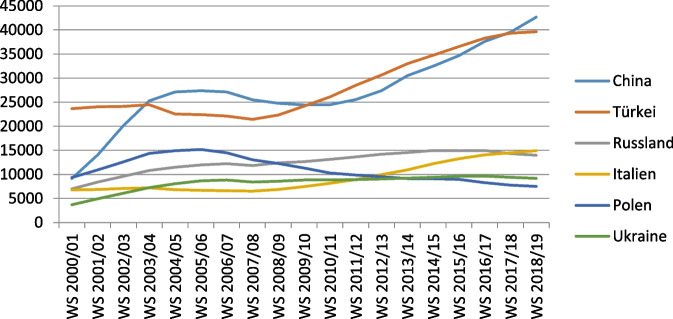
Number of foreign students in Germany (DAAD 2019)

There are 42,676 Chinese students in the 2018/2019 semester, ranking as the biggest group of foreign students in Germany (Statistisches-Bundesamt 2021). Figure 1 illustrates that the number of students from China was similar to those from Poland, Russia, and Italy in the 2000/01 school year – much lower than Turkey as the main source of immigrants to Germany. However, the number of Chinese students has surpassed Turkey in the 2018/19 school year.

Chinese new immigrants in Germany are a diversified group. There were about eighty thousand Chinese new immigrants of Zhejiang origin in September 2020, according to the ninth president of German Federation of Zhejiang Associations, comprising the biggest group of Chinese new immigrants in Germany. According to the President of Wenzhou Association in March 2020, there were about thirty thousand Chinese new immigrants from Wenzhou – the most famous city for its international business in Zhejiang province. There are about twenty thousand Fujian new immigrants in Germany which constitutes the second biggest group. The president of German Federation of Hubei Associations said in May 2022 that there are about three thousand Hubei new immigrants. The remaining population of Chinese new immigrants is from other provinces in China’s Mainland, particularly from Guangdong province, Shanghai municipality, Sichuan province, Shangdong province and Jiangsu province.

What should not be neglected is the various types of associations for Chinese which are considered as one of the three pillars (the other two pillars are Chinese schools and Chinese media) of Chinese immigrants. In Germany, most of the associations have been established since the late 20 century. All German Federation of overseas Chinese was registered in a German court on July 16th, 1998. It was the first national Association of Chinese in Germany after China and Germany officially established diplomatic relations in 1972. Because of the official background of the embassy, the Association has become the head of the associations of Chinese new immigrants, and has led other local associations and two Chinese schools and two Chinese media. These include about fifty region-based Chinese new immigrants associations and trade-based associations. Due to the increase in the number of scholars and students, many university-based associations for Chinese scholars and students have been set up, as well as associations and clubs based on shared interests and hobbies.

According to our survey, the most active associations are region-based associations, in particular, the previous mentioned German Federation of Zhejiang Associations and its twenty sub-associations, including Wenzhou association and Qingtain association. The German Federation of Fujian Associations has a voice in immigrants due to its population and its strong ethnic businesses. There are other types of associations as well, such as the Association of Chinese Gastronomy in Germany, German Chinese Chamber of Commerce, All German Chinese Women’s Federation and the German-Chinese Cultural Exchange Association in Bonn. Catering businesses are the major ethnic economies of Chinese entrepreneurs in Germany. According to the president of the Association of Chinese Gastronomy in Germany: “There are more than 10,345 Chinese restaurants in Germany, and they are employing more than 50,000 full-time employees and 75,000 part-time employees. The total annual turnover of the Chinese restaurant industry exceeds 3.6 billion euro” (Interview, 28 August 2020). Light and Gold (2000:3) define ethnic economy as “any ethnic or immigrants self-employed group, its employers, their co-ethnic employees and their unpaid family worker”. Zhu (2019) argues that Chinese food industry is not only the main foothold of overseas Chinese, but also the growth point of immigrants’ economic capital, social capital and human capital. The occupation diversity of Chinese in Germany is also in present. Since 1980, a considerable number of Chinese have become bus drivers, doctors, nurses, scientists, waiters, musicians, painters, actors, sculptors and writers, and they have worked hard to serve the people of their host countries(Gütinger 1998: 204–205). The population of Chinese had been increased before the COVID-19 pandemic. There is a slight decline comparing the years before the COVID-19 pandemic.

### Global callings and localized initiatives: WeChat for information diffusion and identity making among Chinese new immigrants in Germany

On January 26 2020 – the third day of shutdown in Wuhan, the All-China Federation of Returned Overseas Chinese (ACFROC) published a proposal calling on overseas Chinese to donate to Wuhan. The article was titled as “Proposal of the China Federation of Returned Overseas Chinese on Calling on Overseas Chinese at Home and Abroad to Donate Money and Materials for the Prevention and Control of New Coronavirus Infected Pneumonia”. This initiative was soon posted and forwarded in many WeChat groups worldwide by leaders of associations among Chinese new immigrants. WeChat is the most commonly used communication tool among the leaders of Chinese new immigrant associations in Germany and the offices in China. A normal working procedure is that the leaders read the donation initiative through WeChat, then they confirm the detailed demand for the medical materials needed from the officials of several Chinese governments, most representative the United Front Work Department (UFWD), Federation of Returned Overseas Chinese (FROC), or the Overseas Chinese Affairs Office (OCAO), and then they hold meetings with their fellow members to launch their initiatives to raise money and purchase medical materials. Soon, many organizations of returned overseas Chinese at provincial and municipal levels launched their donation initiatives, aimed at Chinese new immigrants. Wenzhou was one of the cities that issued a global donation initiative on the same day, on January 26 2020.

WeChat plays an important role in the daily life of Chinese new immigrants even though they live outside China. They use WeChat to contact their family members, friends, or colleagues in China. They read news published by the Official Accounts of WeChat. More importantly, they prefer using WeChat even though they have other choices which are more popular in Germany, such as WhatsApp or Facebook. WeChat is an icon through which Chinese new immigrants identify their Chinese fellows in foreign countries. For instance, when two Chinese meet each other in an event for the first time in Germany, normally the first thing they do is to add each other as WeChat friends, rather than getting in contact on WhatsApp or exchanging their German cell phone numbers. Why is this? Castells analyzes the emergence of networked society. “Dominant functions are organized in networks pertaining to a space of flows that links them up around the world, while fragmenting subordinate functions, and people, in the multiple space of places, made of locales increasingly segregated and disconnected from each other” (Castells 1996: 476). How does WeChat affect the networks and societies among the Chinese new immigrants in close contact with distant China?

While keeping close contact with China and trying to make a living in foreign countries, Chinese new immigrants confront with difficulties in adopting to local culture, and thus face some challenges regarding their sense of identity. Taking the Chinese new immigrants in Germany as an example, on the one hand, they are a minority group in terms of population numbers comparing to other German immigrants (Statistical-Bundesamt 2020), such as Muslims and Africans. On the other hand, it is more difficult for Chinese new immigrants in Germany to form a shared cultural identity as Chinese because there are no “Chinatowns” in Germany like there are in America and Britain. However, a shared cultural identity and a sense of community does not necessarily need to form through physical space. As Benedict Anderson (1983) analyzes, human beings have begun to imagine shared communities across physical distance since the invention of publishing techniques and the circulation of written records.

If Anderson’s argument is relevant, we should be able to assume more mechanisms formulating various types of “imagined communities” and shared identities over long distances when the development of information technology has provided tremendous new means of recording and transmitting information. “The work of the imagination, here, consists not in making things up but envisioning something that we cannot see, but which is nonetheless real”(Breuilly 2016). WeChat as a special tool plays an important role among Chinese new immigrants regarding their sense of cultural identity and sense of belonging when they face an identity crisis and the disaster emergency. Barth (1969) writes: “Categorical ethnic distinctions do not depend on an absence of mobility, contact and information, but do entail social processes of exclusion and incorporation whereby discrete categories are maintained despite changing participation and membership in the course of individual life histories”. Barth’s argument is very inspiring in helping us to understand the formation of ethnic identities and relationship among Chinese new immigrants where WeChat plays a crucial role in their everyday lives (Li 2013). Profoundly, Chinese new immigrants have dealt with various lockdown policies and isolation requirements in both countries during the spread of COVID-19 pandemic. The common use of wechat is to keep the Chinese new immigrants communicating to each other and their family members in China. This online connection also contributes to keeping their mental health and to helping them feel safe when, offline meetings have been strictly forbidden.

### Chinese new immigrants’ donations to China: from associations to individuals

In Germany, different types of organizations for Chinese new immigrants actively responded to the donation initiative. The major ones are region-based associations such as the German Federation of Hubei Associations, the German Federation of Zhejiang Associations, the German Federation of Fujian Associations, the Wenzhou Association, the Jiangsu Association, the Beijing Association, the Shanghai Association, and the Guangdong Association. Actually, when Chinese new immigrants heard the news of the outbreak of COVID-19, many donations were sent to China before the global donation initiative.

Wuhan is the capital city of Hubei province. Thus, the German Federation of Hubei Associations was the first association which launched the donation proposal aimed at its fellow members and other associations in Germany on January 23 2020. The president said that they set up two WeChat groups for the donations and received 127,545 yuan (RMB) and 21,944 euros in five days (WeChat interview, March 29 2022). Through contacting with Wuhan officials, the Federation purchased the urgent demanded masks and donated to Huoshen mountain Epidemic Prevention Hospital and Wuhan Central Hospital and the Police Work Support Department of Wuhan Public Security Bureau. On February 14 2020, the German Chinese Chamber of Commerce donated 331 boxes of protective clothing, medical masks and gloves to six hospitals of Hubei Charity Federation and Hankou hospital (IPAMC 2020). On January 26 2020, the Council of the German Association of Chinese Professors (Gesellschaft für Deutsche Professoren Chinesischer Herkunft e.V.) proposed a donation to the hardest hit areas in Wuhan and Hubei. By the end of February, they received 4,532 euros. The money was used to purchase face masks together with the German Tongji Alumni Association, and donated to Wuhan Tongji Hospital, Jinyintan hospital and the Fourth People’s Hospital, and the rest were donated to Huanggang central hospital (German Association of Chinese professors 2020).

This research discovered that associations among Chinese new immigrants have a strong capacity to mobilize their members in Germany for donations, and that the amounts of donations were quite considerable. The case of Wenzhou is worthy of close investigation. According to the offices that received donations in China, both associations and individuals actively participated in the donation scheme. The majority of donations were from various associations. Eleven Chinese new immigrant organizations (including associations and companies) in Germany successfully donated medical materials to Wenzhou by March 2020. Meanwhile, various Wenzhou associations in Italy and Spain contributed significantly to support Wenzhou as well. The calling for donation to Wenzhouese living abroad was issued on January 26 2020. At the end of April, a total of 472 associations/enterprises from forty-five countries had donated to Wenzhou. Donations to Wenzhou included monetary donations of more than 50 million (50,240,044) yuan, more than 14 million (14,602,637) masks, 34.6 thousand (34,603) items of protective clothing, 34.8 thousand (34,752) pairs of goggles, 9 thousand (9,278) thermometers, and more than 3.5 million (3,542,310) items of other supplies (field data, July 24 2020, Wenzhou UFWD).

In addition to donations from associations, individual donations were also very impressive. Many Chinese new immigrants showed their strong emotional attachment to China through their donations. Nineteen retired new immigrants collected 47,000 yuan and donated to Huben Charity Federation in March 2020 (Zhu 2020). An official in Wenzhou UFWD was deeply touched by the donations from an old Chinese man living in Germany:

“At that time, we were quite touched by a doctor living in Germany. […] He donated a lot of money to the Red Cross. When the Red Cross wanted his name to issue him a donation certificate, he signed it ‘anonymous’. […] After adding me as his WeChat friend, he read my posts in my Moment (pengyouquan) about the changing situation of Wenzhou in the pandemic every day. For every Moment about Wenzhou, he put a “Like” and commented. He does not know how to write Chinese characters anymore since he has lived in Germany for many years. But he still loves Wenzhou and China so much.”

The sentimental connection has a strong power in social networks which play a vital role in disaster relief by monetary donation. The donation initiatives from the hometowns of xinyimin in China are targeting at the regional associations globally which motivate many members make contribution accordingly. Commonly, the president of an association contributes firstly and mostly comparing with his or her fellow members. Mr. Hong said since he became the president of Fujain Association four years ago, he has contributed about 300,000 euros. And he not only contributed money to support Fujian against COVID-19 pandemic but also to emergency of members in Germany, for instance, he donated money to support a victim’s families in June 2020. The victim was a fellow restaurateur who committed suicide due to the severe impact of the COVID-19 and its lockdown policies (August 13 2020). The interview with the secretary of Qingtain association shows that it is the entrepreneurs who contribute the most. 160 members out of about 300 members in the WeChat group of Qingtian association donated money (April 23 2020).

### “Global buying” and global transportation: Chinese new immigrants’ capabilities and social networks expansion during the COVID-19 disaster

Overseas Chinese purchased medical materials so actively from January to early March in 2020 all over the world that this became a prominent phenomenon. “Global buying” is used to describe this phenomenon. The statistics of Chinese Customs indicate the large number of medical materials transported from different areas to China.

“From January 24 to February 24, 2020, the national customs inspected and accepted a total of 2.02 billion pieces of epidemic prevention and control materials, worth 6.05 billion yuan. They are 1.63 billion masks, 3.56 million pairs of goggles, 18.62 million pieces of protective clothing, 13.55 million pieces of sterilized items, 4.39 million pieces of medical equipment, and 21.23 million pieces of other epidemic prevention and control materials. Significantly, 16.7% of them are donated medical materials which are valued at 1.09 billion yuan, including 184 million masks, 5.09 million pieces of protective clothing and 1.3 million pairs of goggles” (GACPRC 2020).

People’s Daily (overseas edition) published an article on February 10 2020 to report the process of global buying by Chinese new immigrants.

“They care more about the demand of the home country, forgetting the needs of their own factories. They watched the price increase by 5 times or even 10 times, without blinking their eyes, fearing that they could not buy it. In order to send medical materials to China as soon as possible, they do not hesitate to use high-priced express delivery. They say: ‘regardless of price, fulfilling the domestic demand is our goal’”.

A leader of a group of new immigrants stated the situation in Germany with the author during an interview in June 2020:

“In the act of supporting Wuhan City launched in January, Chinese in Germany actively donated money to support the city. They purchased and transport high-level medical protection materials with a total of more than 1.12 million, effectively supporting the first-line doctors in Hubei Province.”

The authors witnessed the process in person. When they wanted to interview the leaders of associations of Chinese new immigrants in March 2020, many leaders were fully occupied searching for medical materials needed by the hospitals in China, including N95 face masks, protective goggles and disinfectant lotions.

“Global buying” cannot not be accomplished without the success of global transportation because transportation to China was particularly challenging in early 2020, when many flights to China were suspended. Under these circumstances, donors made use of all the methods they could. And Chinese new immigrant businessmen showed their capabilities in logistics to support China in its disaster. Many of them have been doing business in Europe, thus they have established global.logistics chains for their goods before the pandemic. They voluntarily contributed these resources. After purchasing medical materials at very high prices, they stored these materials in their warehouses and transported the materials in their own wagons. Some businessmen persuaded their business partners and put medical materials on the flights of their partners. Some businessmen even persuaded the Chinese government to open a special flight to carry the medical supplies they donated to China. The author witnessed how the leaders of Chinese new immigrant associations in Germany utilized their social networks and business resources to search for medical materials, stored them in their own warehouses and restaurants, and transported them to China. Some association leaders in Germany even drove the goods to the Netherlands to take airplanes in order to send media materials to China as quickly as possible.

Individuals were less powerful in terms of managing resources, but they were just as passionate about delivering medical materials which they had collected to China – e.g., by taking flights, ships, or trains. One of the authors witnessed Chinese in the west and south Germany voluntarily transporting the medical materials they donated to catch a flight from Berlin to Beijing. The officials in Wenzhou were deeply touched by the numerous donations, love, and support from Chinese new immigrants worldwide. They put a red heart on the region where donations were from on a world map; the map was soon full of red hearts. This map was collected by the National Museum of China as a milestone of the anti-epidemic campaign. This map clearly shows the social networks formed by its international new immigrants of Wenzhou city. Crucially, the social networks significantly improving the resilience of the city to control the spread of the virus.

The substantive donations of Chinese new immigrants alleviated the shortage of medical materials in China and enhanced the capabilities of the Chinese government in various levels to deal with the pandemic. The news reports in China about the overseas donations enhanced the confidence of Chinese people to fight against the epidemic. Therefore, the Prime Minister Keqiang Li confirmed the contribution of xinyimin on May 22 2020:

“In Wuhan City and Hubei Province the people have carried on with fortitude and resilience, while people from all sectors of society and our fellow compatriots in Hong Kong, Macao and Taiwan, and overseas have made generous donations, both monetary and in-kind.” (Li 2020)

#### Cultural, economic, and social ties behind social networks

The phenomena of global buying and global donation prove the powerful role of social networks of Chinese new immigrants to support China in coping with the crisis. There are three outstanding driven reasons for this global buying and global donation by Chinese new immigrants. First, there are strong cultural connections of Chinese new immigrants with their motherland and hometowns. As mentioned previously, many Chinese new immigrants are the first or second migrant generations who have daily contact with their families or relatives residing in China. In addition, many Chinese new immigrants still own properties in China, such as houses, apartments, and companies. These properties are often taken care of by their parents, relatives and friends in China. Many Chinese new immigrants go back to China to celebrate Chinese Lunar New Year, and to worship their ancestors in Qingming Festival. They hold strong cultural identity as Chinese. Second, Chinese new immigrants hold close economic connections with China in various ways. Many Chinese new immigrants are merchants running family-based enterprises in Germany, such as selling made-in-China products. They import and export between Germany and China. Many Chinese restaurants in Germany hire Chinese chefs and their fellow villagers and relatives which have shaped the ethnic economy. Travel agencies arranging travel to and from China in Germany work for Chinese tourists. Meanwhile, many of them directly invest in China. Third, there are political connections between Chinese new immigrants and China. In particular, the leaders of organizations work closely with officials in China at the state level (e.g., Consulate General of the People’s Republic of China in Düsseldorf) and the provincial level (e.g., the governments of the new immigrant leaders’ hometowns). Taking Wenzhou’s case as an example, the leaders of new immigrant associations in Germany have stable contacts with the officials of the UFWD, FROC and OCAO in their hometown governments. The above mentioned three aspects form a strong tie between Chinese new immigrants and China, in particular, their hometowns.

Chinese governments at different levels also construct the contact with Chinese new immigrants actively. WeChat is a frequently used tool again. The UFWD in China and the officials of Chinese embassies and consulates built many WeChat groups for the leaders of the new Chinese associations. For instance, the UFWD of Jiangsu province established and maintained friendly relations with more than three hundred Chinese new immigrant associations and more than two thousand key figures in more than one hundred countries and regions (FJROC 2018). Institutionalized social networks have been widely applied in Chinese new immigrants. The field research in Shantou city of Guangdong province discovers the cultural and economic interaction between the hometowns of Shantou emigrants and Chinese associations abroad in August 2022. Associations are the key players of the interaction and conjunction between Chinese immigrants and China while rich businessmen are the leaders of the Chinese association. The hometown of immigrants carefully cultivates the relationship between hometown government and associations of immigrants abroad.

Symbolic and emotional rewards also play a role here. When Chinese new immigrants or the leaders of their associations make outstanding contributions in the work of cooperating with China, they will be invited to attend important ceremonies (e.g., on the National Day of the People’s Republic of China) in Beijing, in their home provinces, cities, or towns, and in the embassy or consulates of China in Germany. For instance, there were about ten Zhejiang people in Germany (six of them from Wenzhou) who were invited to attend the 70^th^ Anniversary on the National Day in Beijing in 2019. Some leaders of Chinese new immigrant associations obtained honor rewards in Zhejiang province and in Wenzhou for their contributions to their hometowns during the COVID-19 pandemic. One association leader was rewarded as a hero in the pandemic war by Zhejiang government in May 2020. The higher a reward is, the higher the capital attained by the awardee. When leaders of Chinese new immigrant associations are awarded by Chinese governments, they take photos and post them in their WeChat Moments and in many WeChat Groups, to show their capacity and share the honor. Their achievements are also reported in detail by Chinese newspapers and social media in Germany. Names of bigger contributors will be written in the local chronicles in their hometown. This also improves the social status of these leaders and associations in Chinese communities in Germany and in their hometowns in China. These kinds of symbolic rewards encourage other leaders to learn from the awardees in future. This is beneficial for obtaining more support from Chinese new immigrants and thus enhancing the capabilities of disaster management, risk reduction and economic development in China. This responds to what Massey (2005)’s argument social network converts it into other forms of capital to improve or maintain their position in society.

Regarding the reasons for the phenomenon discussed above, this research reveals that Chinese new immigrants hold close cultural, economic, and social ties to China through their social networks at various levels. On the one hand, Chinese new immigrants (both individuals and associations) keep close daily contact with people and organizations in China conveniently using internet tools. On the other hand, the Chinese government at different levels also reaches out to Chinese new immigrants actively through different associations (see also Zhuang 2020). Social awards are issued to the leaders of new immigrants. This is an effective reward mechanism to encourage new immigrant leaders to keep close ties with China. Burt (1992) argues that in a social structure of competition, when a person connects two relevant but unconnected individuals/groups, the person has competitive advantages to utilize the resources on both sides. In this case, this person stays in a structural hole, thus in a more advantageous position in his or her institution. According to this structure hole theory, the leaders of various associations are standing in structural holes who have many connections with the nations through ACFROC, UFWD, FROC and OCAO at various levels, thus they can more easily obtain information to launch a proposal and to control most of the resources of the associations. During the donations, the state obtains resources and support from Chinese new immigrants to reduce the risks and then to improves its resilience and capacity to keep people safety. The associations and their leaders received social capital and symbolic capital from the state and Chinese immigrants in return.

### Chinese new immigrants in conflicting understandings on COVID-19 and the support from cultural China

When COVID-19 was gradually brought under control in China in March 2020, it started to spread in other countries outside China. Germany was one of the countries which were seriously affected. Chinese new immigrants in Germany suddenly realized that their status shifted from donors and supporters for China to victims who needed support from China. This situation sheds lights on a new perspective to examine the relation of Chinese new immigrants with the Chinese government during a disaster.

Chinese new immigrants reported that various aspects of their lives – including study, business and everyday life – were seriously affected by the pandemic. When asked how seriously their lives were affected in the survey on a scale of 1 to 10, 39.53% of respondents selected 10 (very seriously). More than ninety percent (90.71%) of respondents selected 5 and above. Almost ninety-eight percent (97.97%) of respondents reported that there were people infected by the virus in the cities in which they lived. Some reported that their friends, classmates, colleagues or family were infected. Regarding the risk of being infected by the virus, about half of the respondents believed the risk was medium or higher. They believed they were not far away from the pandemic and that their health was in danger. They were therefore worried that they would not receive sufficient medical care once they were infected. In this situation, it is not difficult to understand that many Chinese new immigrants wanted to go back to China.

The situation was particularly dire for Chinese new immigrants and students in foreign countries because of the different cultural understandings about the pandemic between China and other countries. The Chinese government and Chinese people regarded COVID-19 as a serious pandemic. Wearing face masks was compulsory from the very beginning of the outbreak. It was not difficult to persuade the Chinese to wear masks because many Chinese still remember the SARS pandemic that happened in South China and Beijing in 2003. Under these circumstances, Chinese people in general had a stronger awareness of the need to wear masks and prevent infection. Chinese hospitals received all infected patients, and the goal was to cure all infected people. China was regarded as much safer than the foreign countries because individuals in China could wear masks to protect themselves, and once they were infected, they would receive good medical care in hospitals.

In contrast, wearing masks was prohibited or at least not encouraged in many foreign countries particularly at the beginning of the pandemic. Many Chinese new immigrants reported in our survey that they experienced discrimination in foreign countries because they wore masks. Moreover, infected cases received medical care in hospital only when the patients were very sick or old. This is why Chinese new immigrants felt their health and even lives were in danger. The fear was particularly strong when Chinese new immigrants and Chinese students compared their lives and situations to those of people living in China. They believed China’s strategies were better and that it was safer staying in China, but they had to stay outside China where government strategies were ineffective.

In order to reduce the uncertainty and improve their capacity to deal with the disaster to protect xinyimin and students, the leaders of various associations decided to expand their social networks to the whole Chinese communities. The best way to achieve their goal was to build a new national association. On March 11 2020, the All-German Epidemic Prevention Working Committee was established, and five leaders of Zhejiang associations and one leader of Fujiang Association were elected to constitute the leadership of the Committee. There were about thirty associations participated in the committee. The committee expanded its connections to its member associations and built strong connections with Chinese consulates and organizations in China. One of the authors worked in the secretary group and saw the committee obtained one million RMB and huge medical material from Zhejiang province and then distributed the money to poor students and immigrants along with various medical materials. The committee successfully expands its networks to region-based associations and other types of associations national wide. For instance, there were 37 temporary offices aimed at dealing with the distribution of health kits as well as any emergency of infection cases around the Bonn City in August 2020. The committee also donated many face masks to German governments, organizations (e.g. police states and old people’s homes) or individuals. This reflects the dynamic of social networks of Chinese new immigrants by institutionalizing their social networks and built offline connections. The establishment of a new organization broadens their social networks by including more associations, even some small marginal associations and many individuals, including scholars, lawyers, doctors, journalists, students and young teenagers of second generation. Actually, the Committee expands its social networks to many weak ties of new immigrants who were not part of any associations, for instance, some female new immigrants who got marry to German. By so doing, the capacity and resilience of the Chinese communities were substantially improved. The achievement of All-German Epidemic Prevention Working Committee was admired by the Chinese communities, Chinese government and German partners.

#### The online and offline support to Chinese new immigrants from China

Chinese governments began to help Chinese new immigrants and students in overseas countries in March 2020. The support was carried out both online and offline. Regarding offline support, health kits were distributed among Chinese new immigrants. The health kits included face masks, N95 masks, disinfectant wipes, Chinese herbal medicine, gloves, and one epidemic prevention manual with positive words encouraging people. In Germany for example, four rounds of health kit distributions were completed near the end of October 2020. Chinese new immigrants and students obtained health kits from Chinese embassies, Chinese consulates or from the Chinese new immigrant organizations. The close connection between Wenzhou and Chinese new immigrants in other countries benefited Chinese new immigrants. As an official in Wenzhou reported to the author:

“As of June 2 2020, the city has donated 1.1007 million pieces of materials, including 1.0579 million masks and 42800 packages of traditional Chinese medicine, with a total value of 2.645 million yuan, to 17 countries (regions) including Italy, Spain, the United States, France, Germany, Kenya, Brazil and Belgium. In addition, as of June 2, 4.532186 million pieces of medical materials were provided by municipal and county governments, and 1.99 million pieces were donated by non-governmental constituted by local youth” (Field data, July 24 2020).

Support was carried out online at the same time. Chinese consulates in foreign countries, such as in Germany and France, invited medical experts in China several times – e.g., the famous Chinese doctor Hongwen Zhang – to online meetings particularly for Chinese new immigrants and Chinese students in Germany (Chinese-Consulate-General-in-Dusseldorf 2020). During the meeting, medical experts explained the development of COVID-19, advised the audience how to deal with different situations when they felt sick or even being infected, and provided students with methods so that they could adjust their psychology and daily schedule to better cope with the mental stress caused by the lockdown during the pandemic. These meetings had multiple effects among Chinese new immigrants and students. Firstly, Chinese new immigrants and students obtained useful and convincing information about the virus and the development of the pandemic. Information provided in these meetings was very valuable to Chinese new immigrants and students who were in a panic because the governments or mass media in foreign countries could not understand the concerns or worries of Chinese new immigrants and students. Secondly, they felt they were not neglected by China since the medical experts joining the meeting were famous doctors with very good reputations explaining the pandemic among Chinese. Chinese new immigrants and Chinese students trusted these experts. Chinese medical experts also suggested that they stay abroad and avoid unnecessary travel across continents.

The official WeChat accounts of Chinese consulates in Germany posted articles regularly at the same time to explain the situation of COVID-19 in different states in Germany and distribute the latest policies from China to overseas Chinese. The advice of not travelling to China unless it was a necessity was also important information delivered to Chinese new immigrants. The communication online was helpful. The survey conducted in this study shows that about eighty-six percent (85.97%) of the respondents took the advice and gave up their plans to travel to China.

Many departments were coordinated in this work. In particular, the UFWD, FROC, and OCAO formulated a series of working principles and procedures for the donation to overseas Chinese with the purpose of providing support on time and improve the resilience of Chinese new immigrants. These departments collaborated and collected medical materials from Chinese governments and non-governmental associations. This research reveals that the UFWD is a key department in this process. Firstly, it represents the state’s policies and intention towards Chinese new immigrants. Secondly, this department effectively investigates the needs of Chinese new immigrants globally by organizing webinars and online meetings for leaders of new immigrant associations. There were connections between government departments in China and the association leaders in overseas countries both before and after the COVID-19 pandemic. WeChat groups were the most frequently used tool. During the pandemic crisis more and more Chinese new immigrant individuals and leaders volunteering to join the WeChat groups built by the department. The officials in Wenzhou mentioned that they had extended their relationship with more leaders of new immigrants during the pandemic. Thirdly, this department coordinates most domestic donations, including docking with airlines to transport anti-epidemic materials, conveying the government’s policies to Chinese new immigrants, and publicizing the government’s anti-epidemic measures. This department also negotiated with the leaders of associations when the leaders donated medical materials to their host countries, such as to Germany.

One of the authors participated in an event when the leaders of two associations donated face masks to the hospital of University of Cologne in Germany. The author could bridge the association leaders and the person in charge in the German hospital because the author knew a Chinese doctor who worked in the hospital. When the author learned the shortage of medical material of the hospital from the doctor, she informed the leaders of Zhejiang association and Jiangsu association. The Zhejiang association first donated 4500 face masks to the hospital which were donated by its members. A month later, another 30,000 face masks were donated to the same hospital with the support of a returnee from Germany. He is a Standing Committee Member of Zhejiang Chinese People’s Political Consultative Conference Committee as well as the Vice Chairman of the FROC in Zhejiang Province. In this donation, the author and the doctor played an important role in connecting the Chinese association and German hospital. However, without the strong ties between Zhejiang associations and the Standing Committee Member in Zhejiang, the donation could not be happened because of the difficulties of cross border logistics and customs clearance in March–May 2020. According to the observation, there were many associations donated masks to the government, universities and supermarkets in several German cities through different networks.

The volunteer donation to the hardest hit county in Germany — the county of Heinsberg was really remarkable. The first infected case of corona-virus in Germany was confirmed in Heinsberg on February 24 2020. In March 2022, according to the county magistrate, Heinsberg neither could obtain enough epidemic prevention resources from the federal government and the state government of Germany, nor could it purchase any materials from the market. In order to control of the COVID-19 spread, German Hangzhou Federation of overseas Chinese, German Qingtian Association and other associations handed over a batch of medical masks, protective clothing and other epidemic prevention supplies to the head of Heinsberg county and the county health bureau to support the county’s epidemic prevention work. This donation was facilitated by the Chinese Consulate General in Düsseldorf. We interviewed the secretary of German Qingtian association who attended the donation on April 23 2022, he said, “We originally had more than 7,000 N95, with a total price of 40,000 euros. After the outbreak of epidemic, the Qingtian association in Germany called on contributions from its fellow members in the name of supporting the fight against the epidemic in hometown. Later, epidemic was under controlled in China, and the FROC in Qingtian informed the association leaders to keep the donation items for overseas Chinese (instead of sending to China). Because the items were medical masks (highly valuable), they were eventually donated to Heinsberg county.” Donations to German institution also reveal the dynamic of social networks of the associations of Chinese new immigrant. The associations work with Chinese consulate in order to have a formal connection with Heinsberg and can donate the medical material in a proper way (reducing risks). During the donations to German society, the goals to reduce stigma and potential discrimination in the host society at that time were achieved. Through civil donation, the trust between China and Germany has been rebuilt.

Many Chinese new immigrants and students who received the health kits took pictures and shared them in WeChat groups or Moments to express their appreciation. The officials of UFWD, FROC, and OCAO also asked the leaders of new immigrant associations in Germany about the details of their work, e.g., when they distributed the health kits to Chinese new immigrants. These kinds of records proof that the associations were working to protect Chinese new immigrants and Chinese students in Germany.

As discussed in this section, the experience of Chinese new immigrants during the COVID-19 pandemic proves that a disaster is a social, cultural, and medical specific issue. The identification of the COVID-19 pandemic is embedded in the social-cultural structure of the Chinese community in Germany. The cultural element also impacts which methods to be chosen to deal with the public health crisis. Actually, how infected people should be treated are questions that are addressed differently in China and in other countries such as the US and those in Europe. Therefore, coping with the pandemic is particularly challenging for Chinese new immigrants because they are caught in between (mainly) two understandings of the pandemic. Some of them wanted to go to China when they were in a panic and their health was in danger. This brought new challenges for China regarding its coping strategies for dealing with the pandemic. Under these circumstances, Chinese governments at different levels made use of internet tools to reinforce established or to construct new online communication channels to support Chinese new immigrants. Medical materials are sent from China to Chinese new immigrants as well. Mobile apps represented by WeChat play crucial roles in this process. This research argues that while understandings of disasters are culturally specific, the solutions to cope with them are culturally specific as well.

## Conclusion

Contributing to existing theories on the social networks of Chinese new immigrants, this paper reveals that social networks of Chinese new immigrants have its strength for combating COVID-19 pandemic for individuals, associations, and for the overall disaster relief in China.

At the micro level, the contribution of follow members supports the donation initiatives of the associations. It shows the strong ties of social connections among members of overseas associations. Meanwhile, members can obtain resources and support from their associations, such as health kits and online courses. At the meso level, the associations can set up new connections to institutionalize weak relationships between associations. The ties between various Chinese associations were rather weak and many even have conflicts with each other before the pandemic. However, during the pandemic, the establishment of All-German Epidemic Prevention Working Committee reflected the resilience of the Chinese new immigrants by reconstructing their social networks and obtaining better social capital by establishing this institution. Furthermore, the associations are key players of social networks of Chinese immigrants based on its well-organized structure and leadership. Associations have a stronger and bigger social network than individuals, and they can cooperate with the home state and host country by joining diverse institutions as a member association or by linking diverse platforms and agencies. As a broker between China and the host country, the Chinese associations prove their capacity and resilience in coping with the COVID-19 crisis. It also shows that the Chinese associations in Europe “need two worlds” (Li 1999b). At the macro level, this research finds that the cultural, economic and social ties have driven the Chinese new immigrants voluntarily to support China in order to reduce uncertainty and to improve resilience and security.

As shown in this study, the experience of Chinese new immigrants shows that the understandings of a disaster and the role of social networks for coping with the disaster are very culturally specific. This research argues that studies on disasters and disaster management should pay more attention to the culture in the host countries where Chinese new immigrants live and Chinese culture, particularly when the fast flow of population and goods worldwide nowadays has connected different regions together tightly. The case study on social networks of Chinese new immigrants in combating the COVID-19 pandemic shows that the processes of reciprocity between Chinese new immigrants and China are beneficial to both sides. The circulation of social capital through social networks builds trust and offers support from and for both sides.

A salient finding of this research is that the wide use of WeChat has become a crucial infrastructure to build the relationship between Chinese new immigrants and their hometowns in China. This is evidenced in several ways. On the one hand, internet tools – represented here by WeChat – serve as an effective means for the communication between Chinese new immigrants and China. This is crucial for both sides to build their resilience and capabilities for dealing with a disaster. On the other hand, internet tools serve to build faith and confidence between two sides by clarifying misunderstandings, transmitting policies from authoritative institutions, reducing rumors, and so on. The cultural, economic, and social ties of Chinese new immigrants to China are all reinforced during the pandemic facilitated by WeChat. The usage of WeChat captures the core cultural characteristics of Chinese internet users within China and beyond in the cyberspace during the global implementation of social distancing and lockdown measures.

## Data Availability

The major data for the article is based on quantitative and qualitative surveys conducted in Germany and China. Some data is from the authories and media.

## Footnotes

1 The survey was supported by the lately established Epidemic Prevention Working Committee in 2020. We appreciate the support of the committee.

2 Besides, the number of Chinese students is increasing in China. According to Wu Ken (2020) “The total number of students studying in Germany exceeds 45,000, making it the largest group of foreign students in Germany. The vast majority of them, about 34,000 people are still staying in Germany” (Lu 2020).
